# Editorial: Pharmaceutically active micropollutants – how serious is the problem and is there a microbial way out?

**DOI:** 10.3389/fmicb.2024.1466334

**Published:** 2024-08-30

**Authors:** Aditi Singh, Japareng Lalung, Irina Ivshina, Irena Kostova

**Affiliations:** ^1^Amity Institute of Biotechnology, Amity University Uttar Pradesh, Lucknow, India; ^2^School of Industrial Technology, University Sains Malaysia, George Town, Malaysia; ^3^Institute of Ecology and Genetics of Microorganisms, Perm Federal Research Center, Ural Branch of the Russian Academy of Sciences, Perm, Russia; ^4^Microbiology and Immunology Department, Perm State National Research University, Perm, Russia; ^5^Department of Chemistry, Faculty of Pharmacy, Medical University-Sofia, Sofia, Bulgaria

**Keywords:** pharmacologically active compounds, PhACs, drug pollution, environmental pollution, bioremediation

Pharmaceutically active compounds (PhACs) like antibiotics, drugs, and hormones are substances, extensively used in agriculture (Nguyen et al., 2023), medicine, and biotechnology (Deb et al., 2019). From 2015 to 2020, global medical dose consumption saw a substantial increase of ~24%, equivalent to 4.5 trillion additional doses (Narayanan et al., 2022). This surge reflects a growing reliance on medications, with significant contributions from populous countries such as India, China, Brazil, and Indonesia. In these regions, it is estimated that more than half of the world's population consumes, on average, over one dose of medicine per person each day (Narayanan et al., 2022). For instance, antibiotic consumption has increased globally in the past two decades (Langford and Morris, 2017; Kovalakova et al., 2020), a lot of which is attributed to improper use, leading to severe surge in antimicrobial resistance (Dadgostar, 2019; Tiseo et al., 2020; WHO, 2024). World Economic Forum in its 2021 report on antimicrobial resistance through water pollution has observed pharmaceutical and industrial waste as one of the four major sources of discharge in water bodies (World Economic Forum, 2021). According to one estimate, a significant portion which can range from 30 to 90% of orally consumed antibiotics are excreted in active form (OECD, 2019). This trend underscores the escalating demand for pharmaceuticals and highlights the importance of managing their environmental impact and ensuring sustainable healthcare practices.

Pharmaceutical production as well as consumption, both lead to discharge of these compounds in water bodies. The primary sources of PhACs in the environment are human and veterinary applications, with major pathways being excretion and discharge through sewage treatment plants (STPs). The presence of pharmaceutically active micropollutants like analgesics (Tyumina et al., 2020), hormones, antifungals (Camacho-Muñoz et al., 2010) along with significant amounts of antibiotics (Singh and Saluja, 2021; Ajibola et al., 2022; Xu et al., 2023; Barathe et al., 2024) being discharged into our environment has emerged as a pressing global concern. These contaminants, originating from various pharmaceutical products, are consistently detected in water bodies, posing risks to ecosystems and human health. Among the most ubiquitous PhACs are non-steroidal anti-inflammatory drugs (NSAIDs) such as diclofenac and ibuprofen (Baratta et al., 2022; do Nascimento et al., 2023; Placova et al., 2023). These substances are widely used for their therapeutic effects but are not fully removed by conventional wastewater treatment processes, leading to their accumulation in aquatic environments (Rastogi et al., 2021; Swiacka et al., 2021; Blasco and Trombini, 2023). For instance, a study by dos Santos et al. (2021) highlighted significant concerns related to human health and environmental risks. Specifically, the synthetic estrogen 17α-ethinylestradiol was found to pose a high risk to human health, while the naturally occurring hormone 17β-estradiol presented a substantial environmental threat. These findings underscore the need for rigorous risk assessments to identify and mitigate the potential hazards posed by these and other chemical contaminants. Apart from groundwater, the contamination of PhACs is also reported in drinking water as well as treated wastewater (Fick et al., 2009; Chander et al., 2016; Kot-Wasik et al., 2016; Gu et al., 2018). In a pioneering systematic study by Bexfield et al. (2019), drinking water from 1,091 sites was analyzed for twenty-one hormones and a hundred and three pharmaceuticals. The study reported the presence of the hormone hydrocortisone, the plastic component bisphenol A, and three pharmaceuticals: carbamazepine, sulfamethoxazole, and meprobamate.

A number of studies have highlighted that the biodegradation of complex pharmaceutical compounds using bacteria, fungi, or their enzymes, is one of the prominent mechanisms for the removal of PhACs from wastewater and the environment (Chen et al., 2022; Habib et al., 2022; Ivshina et al., 2022; Waghmode et al., 2022; Kandasamy et al., 2024). However, in wastewater treatment plants (WWTPs), the removal of stable and low-concentration pharmaceutical compounds primarily relies on adsorption to sludge and biodegradation, whereas in water treatment plants (WTPs), chlorination and the use of activated granular carbon are the most effective methods for removing these compounds (Couto et al., 2019). Additionally, conventional WTPs can reduce but not completely eliminate pharmaceutical compounds from potable water (Couto et al., 2019; Kosek et al., 2020). Some of the gap areas in the efficient removal of pharmaceuticals from water in drinking water treatment plants are—(i) Neglect in design: when designing drinking water treatment plants, the removal of pharmaceuticals is often not prioritized or included as a standard treatment step. This omission is due to several factors, including the primary focus on traditional contaminants like pathogens, heavy metals, and organic pollutants; (ii) Technological and Financial Challenges: incorporating advanced treatment technologies specifically designed to remove pharmaceuticals, such as advanced oxidation processes, activated carbon filtration, or membrane technologies, can be both technologically complex and financially burdensome; (iii) Regulatory gaps: the absence of stringent regulations mandating the removal of pharmaceuticals from drinking water further exacerbates this issue. Without legal requirements, there is little incentive for water treatment facilities to invest in such technologies.

The Research Topic, titled “*Pharmacologically active micropollutants – how serious is the problem and is there a microbial way out?*”, brings to the fore the latest findings on the challenges posed by the many pharmacologically active compounds as pollutants for the environment. It highlights the severity of pollution caused by these compounds, often found in pharmaceuticals and their adverse effects on ecosystems and human health. The research also explores innovative microbial strategies for mitigating these pollutants, presenting microbial intervention as a promising solution to address and reduce the environmental impact of PhACs. This editorial synthesizes the key findings from five significant studies included in our Research Topic, each addressing different facets of this complex issue.

In their review, Tyumina, Subbotina et al. provides an extensive overview of the occurrence of ketoprofen, a widely used non-steroidal anti-inflammatory drug, in aquatic and terrestrial environments between 2017 and 2023. Ketoprofen, known for its persistence and resistance to oxidative metabolism, has been detected in various regions and ecosystems, highlighting its widespread presence and the factors contributing to the entry of ketoprofen into aquatic ecosystems including pharmaceutical waste, improper disposal, and agricultural runoff. It poses significant ecotoxicity risks to vertebrates, invertebrates, plants, and microbes, emphasizing its detrimental impact on these organisms. The breakdown of ketoprofen by fungi, bacteria, and their enzymes as well as phytoremediation techniques and the use of algae to adsorb ketoprofen, are examined, of much significance among them are white rot fungi belonging to *Trametes* spp., *Pleurotus* spp., *Phanerochaete* spp., *Irpex* spp. Authors have previously reported *Rhodococcus erythropolis*, stress-tolerant actinomycetes for ketoprofen degradation (Bazhutin et al., 2022), and preparing a bacterial consortium with this rhodococci was assessed. Overall, the review covers several key points and offers a comprehensive assessment of the negative impacts and consequences of ketoprofen accumulation in ecosystems. It emphasizes the urgent need for developing and implementing effective microbiological remediation strategies to address contamination by such hazardous ecotoxicants.

The review paper by Amobonye et al. provides a comprehensive analysis of how various fungal species can metabolize and degrade PhACs. It is the filamentous fungi of three groups, viz. Ascomycetes, Basidiomycetes, and Zygomycetes, have been most extensively studied, with white rot fungi of Basidiomycota being the most potential example for PhACs breakdown. The study focuses on several fungi, including *Aspergillus luchuensis, Gymnopilus luteofolius, Irpex lacteus, Penicillium oxalicum*, and *Trametes versicolor* which have demonstrated significant potential in breaking down pharmaceutical compounds such as diclofenac, carbamazepine, and ibuprofen. The potential of fungal enzymes in PhAC bioremediation is highlighted, the most explored of them being laccases, peroxidases, and monooxygenases, carrying out detoxification by reduction, oxidation, hydroxylation, dehydrogenation, deamination, dehalogenation, etc. The research elucidates the metabolic pathways employed by these fungi, which facilitate the breakdown of complex pharmaceutical molecules into less harmful byproducts. The mechanism of fungal bioreactors for PhAC degradation is also very comprehensively discussed, emphasizing the need for scalable and sustainable bioremediation solutions. The paper calls for further research into the optimization of fungal bioremediation processes, including the identification of the most effective fungal strains and the environmental conditions that maximize their biodegradative activity.

Gupta et al. in their review provide a comprehensive overview of the current understanding of pharmaceutically active micro-pollutants (PhAMPs) in natural environments. The review examines the factors influencing the pathways and prevalence of these contaminants, highlighting their environmental significance and providing a comprehensive analysis of various methods for removing them from water. The authors critically evaluate traditional water purification methods of physical separation (photolysis, nanofiltration), chemical transformation (reverse osmosis, UV decomposition), and biological methods (biodegradation by microbes), alongside emerging alternative techniques like combinatory treatments and integrative processes for addressing the rising threat of these pollutants. The authors highlight the advantages of using an integrative approach, combining methods such as microfiltration, ultrafiltration, and nanofiltration membranes, along with photo-oxidation using UV light and other advanced oxidation processes. Optimizing factors like membrane selection, reactor design, and operational parameters can enhance these methods' effectiveness. The review also underscores the necessity for detailed, comprehensive, and long-term studies on the health effects of PhAMPs present as water contaminants because such studies can be crucial for formulating regulations and legal standards governing the production and consumption of pharmaceutical products. To combat the escalating issue of pharmaceutical contamination, the review emphasizes the need for innovative therapeutic strategies, like phage therapy, the development of vaccines, and the use of substitutes for natural substances to minimize antibiotic overuse. The review underscores the importance of these new approaches in mitigating the environmental and health risks posed by pharmaceutical pollutants.

The study on phenotypic and metabolic adaptations of *Rhodococcus cerastii* strain IEGM 1243, a bacterium known for its resilience and metabolic versatility by Tyumina, Bazhutin et al., focuses on the adaptive responses of this strain when exposed to two pharmaceuticals. Taking reference to their previous studies on the potential of rhodococci to degrade NSAIDs, paracetamol, and ketoprofen (Ivshina et al., 2019, 2021; Bazhutin et al., 2022), the present study was done to explore the individual and combined effect of diclofenac and ibuprofen. The results were intriguing as *R. cerastii* exhibited moderate biodegradative activity, indicating its potential role in the bioremediation of NSAIDs. Notably, exposure to these drugs resulted in significant phenotypic and metabolic changes within the bacterial cells. For instance, the study observed increased cellular respiration and catalase activity, which are indicative of heightened metabolic processes aimed at neutralizing the toxic effects of the drugs. Morphometric and ultrastructural analysis of *R. cerastii*, when exposed to ibuprofen, showed the structural integrity of cells and the presence of lipid inclusions, polyphosphates, and intracellular membrane-like structures. These adaptations suggest a complex interaction between the bacteria and the pharmaceutical pollutants, highlighting the potential for utilizing such microbial systems in environmental cleanup efforts.

Microbial bioremediation is emerging as one of the most promising techniques for neutralizing and detoxifying pharmaceutically active micro-pollutants. The review article by Saeed et al. provides a comprehensive analysis of the impact of veterinary medicines on the environment, exploring the pathways through which veterinary drugs reach different niches, such as soil and water bodies. Entering through multiple routes, including runoff from agricultural lands, leaching into groundwater, discharge from animal farming facilities, improper disposal of waste, and excretion by treated animals, the paper highlights how these antibiotics, when released untreated into the environment, contribute to the problem of antimicrobial resistance. The review further discusses the traditional methods for eradicating veterinary medicinal effluents and their limitations, such as incomplete removal of contaminants, high costs, and potential secondary pollution. Authors, referring to their previous work (Singh et al., 2022; Singh and Kostova, 2024), have detailed the potential of bioremediation techniques to detoxify pharmaceutical pollutants, examining the mechanisms by which microbes break down veterinary drug compounds, highlighting microbial electrochemical technologies as eco-friendly solutions. The paper emphasizes the importance of optimizing microbial strains, improving bioreactor designs, and developing cost-effective solutions by integrating bioremediation with other treatment methods to achieve comprehensive environmental protection.

The increasing prevalence of pharmaceutically active micropollutants (PhACs) in the environment poses a significant threat to ecosystems and human health. A comprehensive global study by Wilkinson et al. analyzed over one thousand samples from 258 rivers across 137 geographic regions to detect 61 pharmaceutical compounds. The findings revealed that low- to middle-income countries are the most impacted due to inadequate wastewater and waste management infrastructure and pharmaceutical manufacturing practices. Among the compounds detected, carbamazepine, metformin, and caffeine were the most prevalent, found at more than half of the monitored sites (Wilkinson et al., 2022). This underscores the urgent need for improved waste management systems in these regions to mitigate the environmental and health risks posed by pharmaceutical contamination. Therefore, assessing the environmental risks of chemical compounds is crucial to protecting both aquatic and terrestrial biotic systems (Buta et al., 2021; Allel et al., 2023; Aladekoyi et al., 2024). The collection of research within this Research Topic “*Pharmacologically active micropollutants – how serious is the problem and is there a microbial way out*?”, of Frontiers in Microbiology highlights the urgency of addressing this issue and explores innovative microbial solutions. The five papers presented here collectively underscore the critical need for comprehensive strategies to mitigate the impact of PhACs and pave the way for sustainable remediation practices.

The integration of molecular approaches, such as genomics, proteomics, metabolomics, metagenomics, and transcriptomics, collectively referred to as the “Omics approach” has significantly improved our understanding of the metabolic pathways involved in the degradation of organic and inorganic substances during environmental bioremediation (Dangi et al., 2019). Microorganisms possess diverse and unique metabolic capabilities that allow them to assimilate complex molecules for energy, thanks to various processes and enzymes that act on a wide range of substrates. Advances in functional genomics have enabled the manipulation of genes and promoters to enhance these metabolic activities. Genetically engineered microorganisms are more efficient at bioremediation compared to naturally occurring microbes due to their enhanced capabilities in breaking down pollutants. Additionally, synthetic biology, which includes techniques like modifying organisms, creating minimal cells from scratch, designing new artificial functions in cells, and constructing genetic circuits, has further optimized the bioremediation process (Johri et al., 2023). These advancements have collectively made bioremediation more effective in addressing environmental pollution. However, it is equally important to do the ecological safety and risk assessment of the hereditarily and metabolically altered bacteria (Libis et al., 2016; Petsas and Vagi, 2019; Rafeeq et al., 2023).

Barcellos et al. (2022) in their systemic review covering more than 3,000 published documents from 1990 to 2020 on the management of pharmaceutical micropollutants and after analyzing various technical and governmental macro-to-micro approaches, have concluded that programs like drug take-back, and community sensitization on drug use reduction or proper disposal of discarded medicines, can be effective methods to better manage these pollutants and reduce environmental burden. They have given fifteen opportunities for developing countries which can be promising in bringing down PhACs pollution in urban waters. There should be strict norms for pharmaceutical compound use. Strict legal norms could help regulate and control the prescription and consumption of antibiotics, ensuring they are used only when necessary and in appropriate dosages; regulated use of antibiotics in agricultural and veterinary medicine, only for treating infections and not as growth promoters; following international standards and their strict compliance. To achieve these standards, there is a need for regular surveillance and robust monitoring programs for pharmaceutical residues in water bodies, soil, and food products to detect and quantify these contaminants at low concentrations. Advanced detection technologies utilizing advanced analytical techniques, such as mass spectrometry and high-performance liquid chromatography, can improve the accuracy and sensitivity of monitoring efforts and will be able to detect even trace amounts of pharmaceuticals. Systematic data collection and analysis help in understanding the sources, distribution, and fate of pharmaceutical contaminants. This information is critical for developing effective mitigation strategies and informing policy decisions. Lastly, ensuring transparency in monitoring results and raising public awareness about the potential risks of pharmaceuticals in the environment can drive community support for regulatory measures and encourage responsible pharmaceutical use and disposal practices. Thus, by addressing these points, we can work toward a more comprehensive and effective approach to managing pharmaceutical contaminants in the environment, protecting public health, and preserving ecosystems.
